# Mechanical Behavior of Selective Laser Melting (SLM) Parts with Varying Thicknesses in a Saline Environment under Different Exposure Times

**DOI:** 10.3390/ma17091959

**Published:** 2024-04-24

**Authors:** Maaz Akhtar, Muhammad Samiuddin, Muhammad Muzamil, Muhammad Ali Siddiqui, Rashid Khan, Naser A. Alsaleh, Ali Khursheed Siddiqui, Joy Djuansjah, Arfan Majeed

**Affiliations:** 1 Mechanical and Industrial Engineering Department, College of Engineering, Imam Mohammad Ibn Saud Islamic University, Riyadh 11432, Saudi Arabia; rakhan@imamu.edu.sa (R.K.); naalsaleh@imamu.edu.sa (N.A.A.); aksiddiqui@imamu.edu.sa (A.K.S.); jrdjuansjah@imamu.edu.sa (J.D.); 2 Metallurgical Engineering Department, NED University of Engineering & Technology, Karachi 75270, Sindh, Pakistan; engr.sami@neduet.edu.pk (M.S.); m.siddiqui@cloud.neduet.edu.pk (M.A.S.); 3 Mechanical Engineering Department, NED University of Engineering & Technology, Karachi 75270, Sindh, Pakistan; muzamil@neduet.edu.pk; 4 School of Mechanical Engineering, Northwestern Polytechnical University, Xi’an 710072, China; amajeed@mail.nwpu.edu.cn

**Keywords:** selective laser melting (SLM), AlSi10Mg, corrosion behavior, mechanical properties, mass loss, NaCl solution, exposure time

## Abstract

A promising method for additive manufacturing that makes it possible to produce intricate and personalized parts is selective laser melting (SLM). However, the mechanical properties of as-corroded SLM parts are still areas of concern. This research investigates the mechanical behavior of SLM parts that are exposed to a saline environment containing a 3.5% NaCl solution for varying lengths of time. The exposure times chosen for this study were 10 days, 20 days, and 30 days. The results reveal that the tensile strength of the parts is significantly affected by the duration of exposure. Additionally, the study also examined the influence of porosity on the corrosion behavior of the parts. The analysis included studying the mass loss of the parts over time, and a regression analysis was conducted to analyze the relationship between exposure time and mass loss. In addition, the utilization of scanning electron microscopy (SEM) and X-ray photo spectroscopy (XPS) techniques yielded valuable insights into the fundamental mechanisms accountable for the observed corrosion and mechanical behavior. It was found that the presence of corrosion products (i.e., oxide layer) and pitting contributed to the degradation of the SLM parts in the saline environment. This research emphasizes the importance of considering part thickness in the design of SLM components for corrosive environments and provides insights for enhancing their performance and durability.

## 1. Introduction

Aluminum alloys are generally produced by forging [1], casting [2], and assembling [3,4]. These fabrication processes are not capable of producing complex geometries and fine micro-lattices [5]. However, additive manufacturing (AM) from layered materials makes it possible to produce intricate products [6]. Liu et al. investigated advanced design considerations for AM, emphasizing the role of lattice structures in enhancing mechanical performance [7]. Javed and Haleem explored the application of AM in the medical field, specifically in the fabrication of customized implants for improved patient outcomes [8]. Dzugan et al. investigated the impact of process parameters about the mechanical properties of AM-produced parts, highlighting the importance of optimization for enhanced performance [9]. Bandyopadhyay and Heer addressed the challenges of AM relating to material compatibility, offering insights into material selection for improved functionality [10]. The environmental sustainability of AM processes is a critical aspect. Gao et al. discussed eco-friendly materials and recycling methods in additive manufacturing [11].

With its integration of melting technology, powder bed fusion (PBF) is currently one of the most popular AM techniques for producing metallic components [12,13]. It has given rise to several subcategories, such as electron beam melting (EBM), direct metal laser sintering (DMLS), selective laser sintering (SLS), and direct metal melting (SLM). Therefore, laser powder bed fusion (LPBF), and most often SLM, is the derived formal term used whenever a high-power laser source is used for melting [14]. 

Amongst different AM techniques, SLM is one of the best techniques that have the ability to create three-dimensional parts by a layer-to-layer process. SLM offers unparalleled design flexibility and can manage structural complexity [15]. SLM is used in a number of industries, including consumer goods, aerospace, and automotive. While this technology is still gaining pace, one key issue that needs to be thoroughly investigated is mechanical behavior, particularly in environments where corrosive agents like saline solutions are prevalent [16,17,18,19]. Regarding the mechanical behavior of parts produced by SLM, a variety of factors are involved, including microstructural characteristics revealed by the layer-by-layer deposition process and residual stresses, which have the power to significantly alter the performance of the material [8,20].

Both the manufacturing process and design parameters have a significant impact on the mechanical properties of lattice structures produced using the SLM process. Depending on the application requirements, different design approaches (such as generative design, topology optimization, and biomimetic) to lattice structure design are used [21]. Tobah et al. [22] discussed the use of various commercial powders in the LPBF technique to produce duplex stainless steel having better mechanical properties. Two crucial elements of their investigation were the choice of laser powder and scan speed. They used the dislocation motion, nanoscale precipitation, and grain size to assess tensile behavior. Improved mechanical characteristics of as-printed alloys were observed, with scan speed serving as a key motivator [23]. Another efficient way to create lattice structures is to use deep learning (DL) algorithms [22,24]. A thorough investigation of the effects of various processing factors on relative density, porosity, surface roughness, and dimensional correctness was provided by Nandhakumar and Venkatesan [25]. Similarly, it has been observed by Chandpasha and Apparao that a single SLM parameter can lead to markedly different printed part microstructural and mechanical properties. Understanding how the various SLM process parameters affect the part quality and characteristics is therefore crucial. Aluminum–silicon-based alloys (Al-Si), namely AlSi10Mg, AlSi12, A356 (AlSi7Mg0.3), and A357 (AlSi7Mg0.7), are among the several alloy combinations that have been widely utilized in the SLM process because of their fabricability [26].

It should be mentioned that few aluminum series have poor SLM fabricability, such as 2xxx. The manufacturing process parameters that were employed in those items were crucial. For SLM parts made of aluminum, Liu et al. investigated various combinations of laser powders and scan speeds. They found the optimal values at samples with a laser powder of 360 W at 1150 mm/s scan speed. On samples, they used solid-solution treatment and direct aging to investigate the aging process. The solid-solution treatment was reported to exhibit greater hardness and also showed accelerated aging [27]. 

The corrosion behavior of SLM parts is an important factor to consider when evaluating the structure integrity and longevity of additively manufactured components. The unique microscopic features and residual stress introduced by SLM can considerably alter the corrosion resistance qualities of such materials. The saline environment can cause corrosion in materials that are used in a wide range of applications, from medical implants to nautical components. The way these elements interact with the corrosive media practically becomes quite important [28]. The mechanical behavior of SLM parts might deteriorate due to corrosion in these types of applications, which poses a serious risk to the reliability and integrity of the components that are created. A search through the existing literature indicates a vast array of research focusing on the corrosion properties of these SLM samples across various metals, such as alloys, and in various types of environmental settings. Suryawanshi et al. delved deep into the research to learn more about the resistance to corrosion for stainless steel made through SLM. They closely examine the impact of microscopic flaws as well as any post-processing treatments [29]. Numerous studies on titanium-based alloys have also been carried out by different researchers [30,31]. These studies have also shed light on the important roles that alloy composition and heat treatments play in this process. Tshephe et al. discussed the problem of corrosion in different titanium alloys and discussed remedies to improve the corrosion behavior of titanium alloys produced by SLM [30]. In addition, the study results of Yang et al. [32] and Vrancken et al. [33] all contribute to expanding our knowledge of the mechanisms underlying corrosion in SLM samples by considering variables like the properties of the powder and the real laser processing settings. One of the most often utilized alloys of aluminum that integrates with SLM and shows good mechanical behavior in as-built settings is AlSi10Mg [34]. Numerous aluminum alloys made by AM, such as AlSi10Mg, have potential uses in the aerospace and automotive industries under both static and dynamic conditions [35]. In these and many other applications, the environment is harsh, and the temperature might occasionally rise when working for extended periods of time. Additionally, the likelihood of corrosion is raised. This may have an impact on the component’s mechanical behavior, leading to more noticeable precipitate dissolution and coarsening [36].

The present study explores the complex relationship among the mechanical characteristics of SLM components, alterations in their thickness, and their exposure to a saline environment over different time intervals. This information is essential for enhancing designs and assuring these components work over a broad spectrum of applications. Additionally, analyzing the consequences of prolonged contact to a saline environment provides crucial information regarding the durability and corrosion resistance of those manufactured SLM parts. 

## 2. Materials and Methods

**Figure 1:**
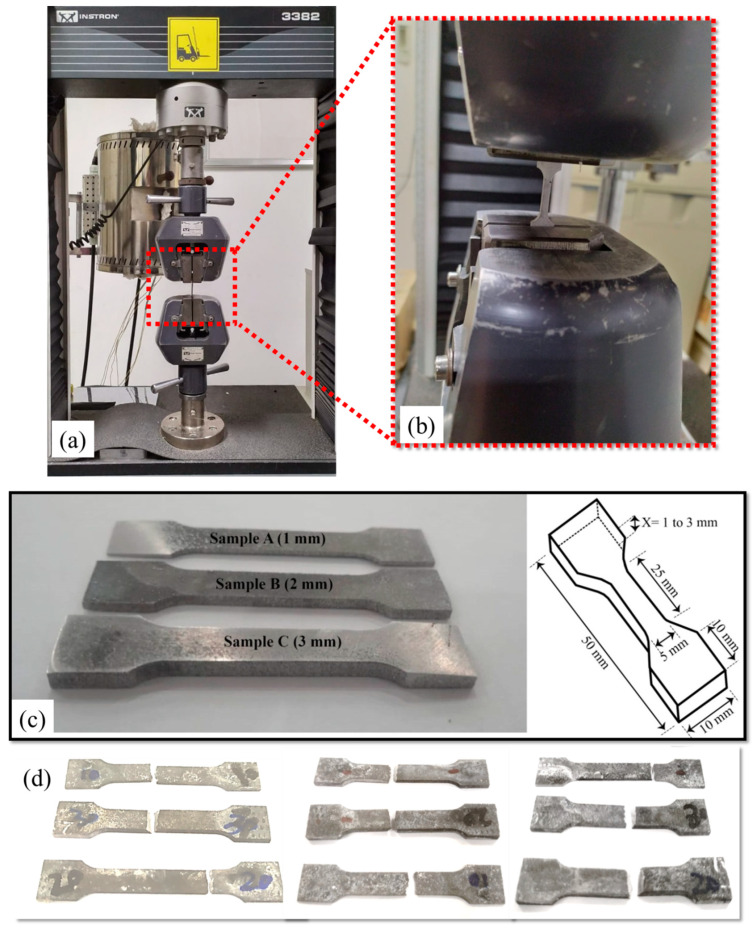
Experimental details for tensile testing: (**a**) testing machine, (**b**) sample’s placement, (**c**) as-prepared tensile specimens along with the sample’s dimensions, and (**d**) specimens after tensile testing.

In this study, the optimal process parameters from the authors’ preliminary studies were used [37]. The experimentations involved three different wall thicknesses of SLM parts that were used to create rectangular samples for various testing purposes. The samples were manufactured in a vertical orientation along the z-axis and were cut from a substrate using wire electrical discharge machining. The testing involved subjecting the samples to tensile forces according to the E8/E8M sub-scale also shown in Figure 1. The powder used for the preparation of SLM parts was sourced from the Powder Alloy Corporation (PAC) based in USA. The author has previously published papers focusing on the optimization of processing parameters. For detailed information regarding the powder morphology and size distribution, readers are referred to reference [38], which provides the relevant details of and insights into the powder characteristics used in the SLM process. A checkerboard scanning strategy with an angle of 67° was employed during the fabrication process. Before the powder was used for fabricating the specimens, it was dried in a drying oven at 70 °C for 4 h. For the fabrication of the test specimens, an SLM 280HL machine manufactured by SLM Solutions in Lübeck, Germany was employed. The parameters were optimized based on the authors’ previously published work [39,40], and the specific details can be found in Table 1. 

Firstly, the corrosion behavior of the thin-walled specimens was assessed by immersing samples of different thicknesses in a 3.5% NaCl solution for varying exposure times i.e., (10, 20, and 30 days). This allowed for the examination of their performance under corrosive conditions in terms of their mass loss. Following the corrosion process, the samples were cleaned in accordance with the ASTM G1-90 standard [41]. The cleaning procedure involved immersing the samples in a freshly prepared solution comprised of 50 mL of phosphoric acid, 20 gm of chromium trioxide (CrO_3_), and reagent water to make a total volume of 1000 mL. The immersion in hot water at a temperature of 90 °C was carried out for a duration of 5 to 10 min. Subsequently, the cleaned samples were weighed using a precision weighing balance with an accuracy of 0.0001 gm. The mass loss was determined by calculating the difference in weights before and after the corrosion process, expressed as a percentage. The data collected were recorded by averaging the results of three samples.

After this, the mechanical properties of the specimens were evaluated for all as-corroded samples. Scanning electron microscopy (SEM) analysis was conducted using a Zeiss Supra55 scanning electron microscope system (Oberkochen, Germany) to examine the fracture surface morphologies. XPS analysis was also conducted for a better understanding of the corrosion behavior.

Tensile testing was performed on the specimens using a universal Instron-3382 testing system equipped (Norwood, MA, USA) with an extensometer and a 100 kN load cell, enabling the assessment of their mechanical properties at room temperature. The ASTM standard E8/E8M [42] sub-scale samples were obtained by wire cutting from the substrate, and the tests were conducted at a crosshead speed of 0.50 mm/min. Additionally, microhardness measurements were carried out using an LECO AMH-43 automatic hardness tester with a Vickers indenter (St. Joseph, MN, USA), and the average microhardness values were determined based on 10 measurements of each specimen. This comprehensive analysis provided insights into the fracture behavior, tensile properties, and hardness characteristics of the thin-walled specimens after exposure to corrosive environments with different immersion times and various thicknesses.

## 3. Results and Discussion

### 3.1. Corrosion Behavior of SLM Parts in 3.5% NaCl Solution

**Figure 2:**
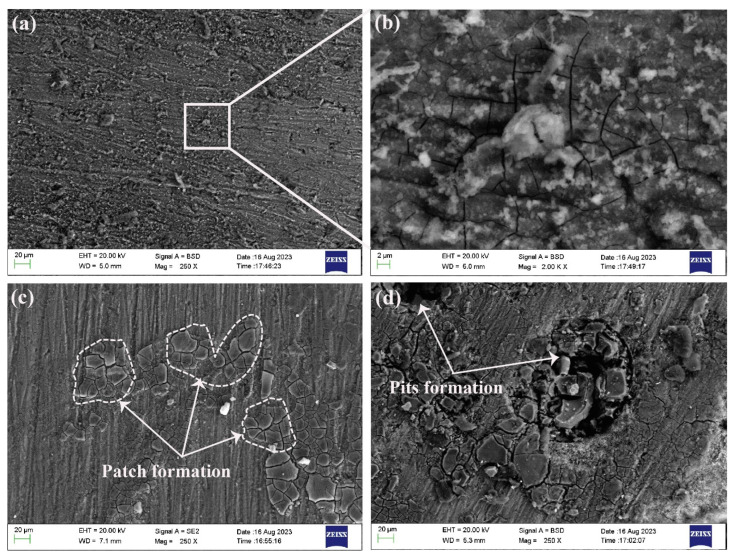
SEM images of the corroded surfaces of sample C: (**a**) 10-day exposure time, (**b**) magnified image of (**a**), (**c**) 20-day exposure time, and (**d**) 30-day exposure time.

The surface examination was performed using the SEM for all the samples. Representative SEM scans of the studied samples are shown in Figure 2. It is shown that progressive deterioration occurs with the increase in exposure time, as is evident from the surfaces. In the study, a magnified image specifically for the 10-day exposure period was included. This was performed to emphasize and examine the early stages of surface corrosion, as well as to highlight the visibility of cracks in the magnified image. By focusing on the 10-day period, the researchers aimed to provide a comprehensive analysis of the initial corrosion behavior, the surface morphology, and the presence of cracks in the sample.

Figure 2a presents the sample after a 10-day exposure period, where the surface of the sample displays a noticeable deposit layer consisting of corrosion products, which can be clearly observed. This deposit layer indicates the initiation of corrosion. To provide a closer look at this deposit layer, Figure 2b presents a magnified area of interest, allowing for a more detailed examination and analysis. The magnified image provides valuable insights into the characteristics and composition of the deposit layer, contributing to a better understanding of the corrosion process in the early stages. Figure 2a presents the sample after a 10-day exposure period. The surface of the sample displayed a noticeable deposit layer consisting of corrosion products, which can be clearly observed. In Figure 2b, a magnified area of interest provides a closer look at this deposit layer.

Corrosion in a saline environment can occur through various electrochemical processes, including the formation of galvanic cells and the initiation of localized corrosion. These processes can lead to the breakdown of the alloy’s protective oxide layer [41,43], exposing the underlying material to further degradation. When SLM AlSi10Mg parts are subjected to prolonged exposure to a 3.5% NaCl solution, the corrosive attack gradually affects the material’s microstructure. The chloride ions present in the solution can penetrate the alloy’s surface, causing the formation of corrosion products and accelerating the breakdown of material [44,45].

As the corrosion progresses, the AlSi10Mg alloy may undergo the selective dissolution of its constituent elements, particularly aluminum and magnesium [45,46]. This dissolution can result in the formation of pits and crevices (see Figure 2d) on the surface of the parts, leading to localized corrosion and a decrease in mechanical properties.

The process of the dissolution of aluminum and its subsequent re-passivation can be explained by the following chemical reactions that occur on the surfaces of SLM parts [47]. Equation (1) represents the oxidation reaction that takes place at the anode during the anodic dissolution of the aluminum alloy. Similarly, Equation (2) represents the reduction reaction that occurs at the cathode during the process of oxygen reduction. These two reactions are integral to the overall electrochemical process that happens during the corrosion of AlSi10Mg SLM parts. The anodic dissolution of aluminum results in the formation of aluminum ions, while the reduction in oxygen at the cathode consumes electrons and generates hydroxide ions [48,49].

Al → Al^3+^ + 3e^−^ (1)

O_2_ + 2H_2_O + 4e^−^ → 4OH^−^ (2)

Al^3+^ + 3OH^−^ → Al(OH)_3_ (3)

Furthermore, the initiation of small cracks is evident, indicating the spalling or detachment of the outer layer. These small cracks can be attributed to the dehydration process that occurs during drying. As a result of this process, the hydrated form of aluminum undergoes a transformation to Al_2_O_3_ [41,49], leaving behind these cracks on the surface also confirmed by XPS analysis and Figure 2a. When a Cl^−^ concentration is present, the anodic reaction can be described by the following equations:

Al(OH)^2+^ + Cl^−^ → Al(OH)Cl^+^ (4)

Al(OH)Cl^+^ + H_2_O → Al(OH)_2_Cl + H^+^ (5)

Increasing the concentration of Cl^−^ causes Reaction (Equation (4)) to accelerate, leading to the continuous consumption of the intermediate product Al(OH)^2+^ [50]. As a result, the anodic reaction of the Al matrix speeds up. The intermediate corrosion products formed have the ability to adhere to the alloy, which immediately triggers the formation of a thick and protective hydroxide layer on the surface of the alloy.

Additionally, as the exposure time increased, the small cracks that were initially observed underwent a further transformation and grew into larger cracks. This progression can be seen in Figure 2c, where the surface layer displays noticeable patches. Over time, these small patches evolved into pit formations, as depicted in Figure 2c, when the exposure period reached 30 days. The presence of cracks in the material facilitated the formation of loose blocks, which were prone to detachment. These cracks continued to propagate along existing microcracks, posing an increased risk of structural damage and fragmentation within the material after longer exposure times (specifically, 20 and 30 days), as also evident in Figure 2c,d.

#### 3.1.1. Mass Loss with Different Exposure Times in 3.5% NaCl Solution

**Figure 3:**
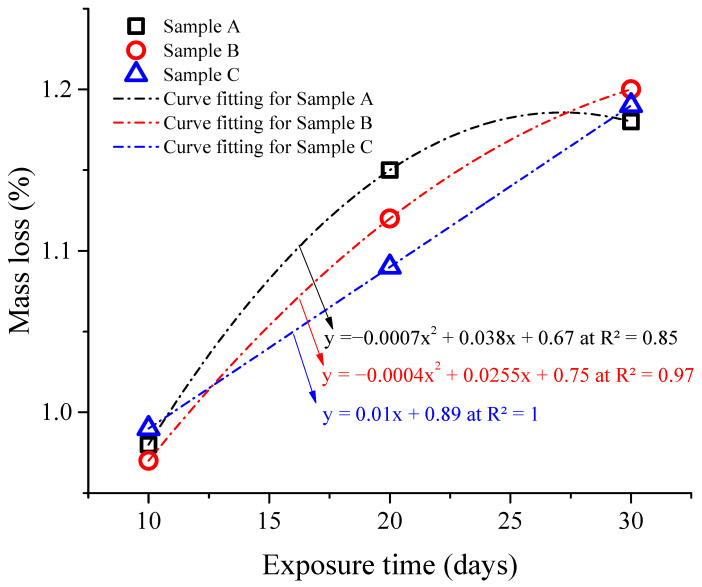
Change in mass after immersion in 3.5% NaCl solution with different exposure times.

The corrosion test involved analyzing the mass changes in the samples at different exposure times, as depicted in Figure 3. The data reveal that, as the exposure time increases, the samples exhibit greater mass loss. However, there was no apparent relationship observed between the thicknesses of the samples and their respective mass losses during the corrosion process in a 3.5% NaCl solution.

Specifically, sample A experienced mass losses of 0.98%, 1.15%, and 1.18% after 10, 20, and 30 days of exposure, respectively. Sample B exhibited mass losses of 0.97%, 1.12%, and 1.2% over the same exposure periods. Sample C, on the other hand, showed mass losses of 0.99%, 1.09%, and 1.119%. Sample A had a thickness of 1 mm, while Sample B and Sample C had thicknesses of 2 mm and 3 mm, respectively. Despite these variations in thickness, no discernible relationship between thickness and mass loss during the corrosion process was observed in the 3.5% NaCl solution. 

Moreover, the resulting data led to the formulation of three equations, each representing the mass loss (Y-axis) against exposure time (X-axis). Regression analysis was performed to establish the mathematical relations between mass loss and exposure time. The data were well fitted with R^2^ values of over 85%, as shown in Figure 3. Since the equations are formed by mass loss as the Y-axis and exposure time as the X-axis, we can compare the slopes of the equations to understand the corrosion loss behavior. Upon analysis, it was observed that the equation representing sample C displayed a linear relationship with a constant slope. This suggests a consistent corrosion rate over time, indicating that the corrosion loss remains constant. For samples A and B, the equations exhibited a curve with a decreasing slope. However, it was noted that the curve for sample A had a steeper inclination towards the X-axis compared to sample B. This implies a higher initial corrosion rate that gradually decreases over time. Therefore, based on the comparison of the equations, it can be concluded that the equation representing sample A corresponds to a higher corrosion loss compared to the equations for samples B and C.

#### 3.1.2. Effect of Developed Porosities on the Corrosion of SLM Parts

**Figure 4:**
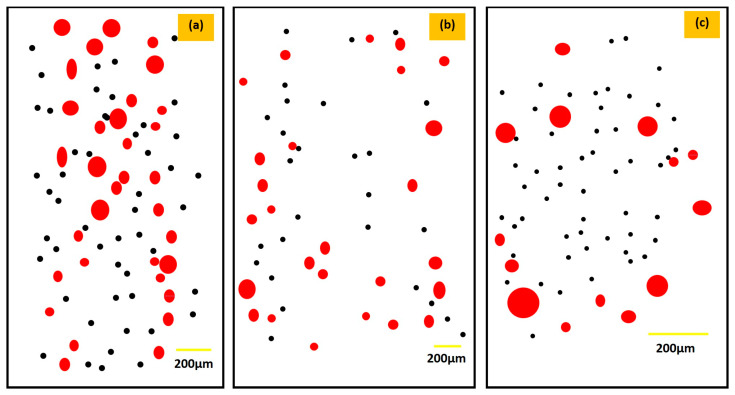
Illustration of porosity development on the as-built SLM sample (before corrosion); (**a**) for a thickness > 1 mm, (**b**) for a thickness > 2 mm, and (**c**) for a thickness > 3 mm.

The outcomes regarding corrosion and mass losses are presented in Section 3.1 and its subsection, Section 3.1.1. These results are specifically highlighted in Table 2, which covers three different thicknesses. The results obtained for the three different thicknesses can be assessed and described under the sure presence of inherited available porosities at the surface, which comes in direct contact with the saline environment. In addition, the above results indicate a noticeable relation with the increase in thickness possibly due to any improvement in the porosities at the exposure surface. Hence, it is expected that the corrosion properties will be influenced accordingly. For this, a certain experimental study is required to provide information on the variation in the porosities while printing different thicknesses through the SLM process. Zhang et al. [39] provided a concrete blueprint by providing the variation in porosities at the same parameters of SLM. Based on this, a schematic illustration is provided in Figure 4, which highlights the variation in the porosities while printing the three relevant types of thicknesses concerning this work.

The illustration is developed by the available porosities at the top surface of the printed samples in the as-built condition, which will come into contact with the saline environment. As highlighted in the study [39] and illustration (Figure 4), more porosities can be observed in the thin-wall sample, while the porosities continue decreasing with the increase in the wall thickness due to the optimum condition developed for the melting and solidification for the powder, and the full penetration of the laser beam develops fine bonding among the particles and the grains. 

All three scenarios are illustrated in Figure 4a–c, where the printing of the sample (thickness > 1) in Figure 4a shows a higher occurrence of porosities compared to the illustration provided in Figure 4b,c for thicknesses greater than 2 mm and 3 mm, respectively. As shown in Figure 4a, the massive pockets of large porosities (highlighted in red) and small porosities (highlighted in black) highlight the high-density occurrence of porosities concentrated at the center. However, the low-density occurrence of porosities is clearly depicted in the illustration in Figure 4b,c when the wall thickness increased to 2 mm and 3 mm due to sufficient bonding and reduction in the pores’ development. Furthermore, both the large and small porosity densities decreased, and specifically, large porosities became concentrated at the edge, as shown in the illustration in Figure 4b,c. 

Therefore, to make a relation of the three selected thicknesses provided in Table 2 with the above illustration in Figure 4, the porosity sizes, probably in samples A and B, could range from 25 to 85 microns, where sample B displays smaller pore sizes, specifically below 50 microns [39], as also illustrated in Figure 4b. Conversely, sample C exhibited minimal pore presence in contrast to samples A and B, according to the illustration presented in Figure 4c. The higher occurrence of pores in the samples led to an increased surface area susceptible to corrosion, resulting in a more pronounced corrosion response. This correlation of porosities is attributed to factors such as the proper melting and solidification of powder particles, effective penetration of the laser beam, and the development of strong bonding between particles and the solidified grain. In the case of samples A and B, the limited time available for melting and solidification resulted in the formation of extensive porosity pockets. 

The illustration reveals a significant reduction in porosities as the thickness increase from 1 mm to 3 mm, attributable to enhanced bonding, which leads to decreased pore formation and gas entrapment. This effect was also evident in the steeper curve of the equation representing sample A, indicating a higher corrosion loss over time, as depicted in Figure 3. Therefore, it can be deduced that the increased corrosion in sample A was primarily influenced by the presence of porosity, which manifested through the steeper slope of its equation.

#### 3.1.3. XPS Analysis of as-Corroded SLM Parts

**Figure 5:**
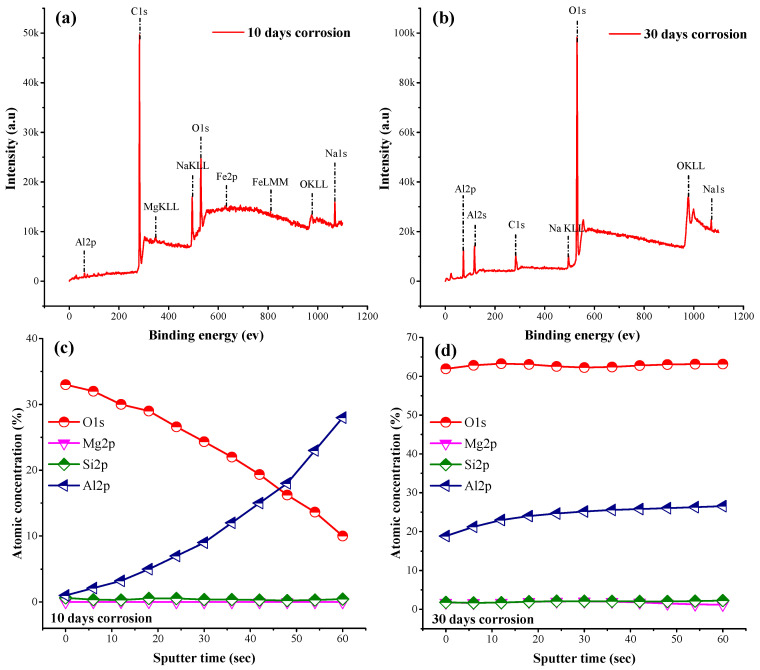
XPS survey scans of the corroded surfaces with different exposure times: (**a**) 10 days, (**b**) 30 days, (**c**) XPS profile of the corroded surface with 10-day exposure time, and (**d**) XPS profile of the corroded surface with 30-day exposure time.

After conducting corrosion experiments with different exposure times, the samples surface were analyzed through X-ray photoelectron spectroscopy (ULVAC-PHI, Inc. Chigasaki, Kanagawa, Japan PHI 5000 VersaProbe III). According to the XPS analysis in Figure 5, the sample’s corroded surface primarily consist of aluminum, oxygen with carbon, sodium, and chlorine contaminations present in the 3.5% NaCl solution. Figure 5a shows survey scans of the as-corroded SLM part with a 10-day exposure time. It indicates the presence of characteristic peaks of Al2p, MgKLL, Fe2p, and O1s elements. Similarly, survey scans of a 30-day corroded SLM surface was also examined and is displayed in Figure 5b. Notice that the Mg element was not detected due to its lowest ppm levels. Possibly, pits were formed due to this selective dissolution. Mg elements are also evident in the SEM images (see Figure 2). This indicates that Mg was dissolved during the corrosion process under the action of Cl^−^ ion activity [51,52,53]. Additionally, it can be easily grasped from the two scans that the O1s peak intensified for the samples treated with a 30-day exposure time. Thus, the peak intensity of O1s shows that the surface becomes oxidized with the depletion of Mg content. From the analysis of the survey scans (i.e., Figure 5a,b) of the two samples, it can be concluded that the corrosion product that formed on the surface is mainly composed of Al and O, with some minor traces of residual elements of the electrolyte solution [43,53,54]. 

In order to determine the composition of the passive film formed during the corrosion process, profile scans were also executed on the same samples. Figure 5c,d represent the scans obtained after Ar-sputtering of the corroded surface with a depth profiling rate of 7 to 12 nm/min. Figure 5c illustrates that the surface oxidation of the sample treated for 10 days results in a high oxygen content. Furthermore, the presence of oxide is not eliminated within the layer, suggesting that it may exist at a lower depth than the surface. From the depth profile, it can be seen that the amount of oxygen decreases whereas aluminum concentration increases within the studied sputtering time. Similar depth-profile scanning was performed on the sample with a 30-day exposure time shown in Figure 5d. The results indicate the presence of stable Al- and O-based films that remain on the surface, even after the subsequent sputtering operation. Furthermore, no compositional variation was noticed in the stipulated sputtering time, which suggested the formation Al_2_O_3_ also evident from the survey scans, which is discussed in the subsequent section.

**Figure 6:**
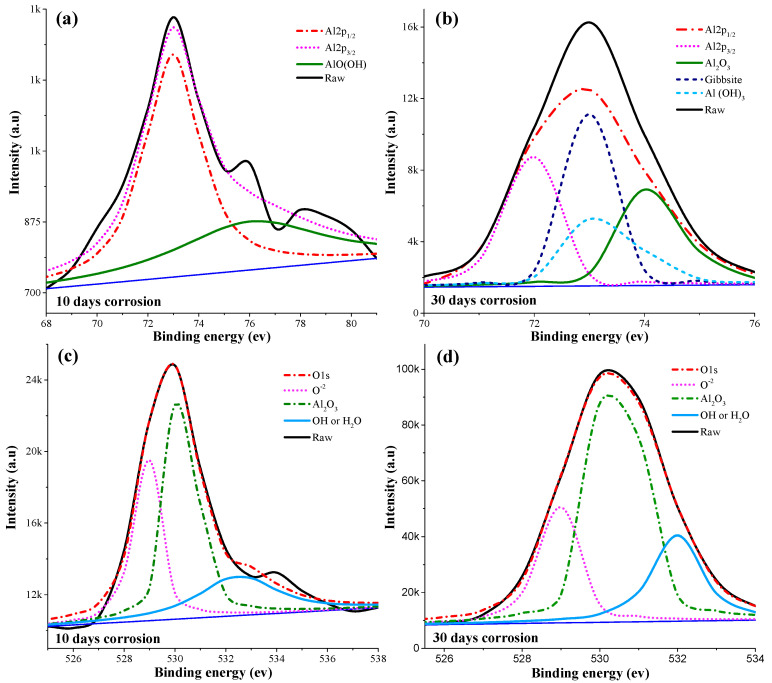
Deconvoluted XPS scans of the corroded surface after immersion in 3.5% NaCl solution: (**a**) Al2p with 10-day exposure time, (**b**) Al2p with 30-day exposure time, (**c**) O1s with 10-day exposure time, and (**d**) O1s with 30-day exposure time.

Figure 6 presents the deconvoluted high-resolution peaks of Al2p and O1s obtained after peak fitting through multipack V 9.9.2 software^®^. In Figure 6a,b, the Al2p spectra of the samples are displayed, providing information about the aluminum oxide contributions on the surface. The dominant aluminum oxide species identified is Al_2_O_3_, which was observed at binding energies of 72.9 eV and 75.0 eV [55,56]. These peaks correspond to the characteristic binding energies of Al_2_O_3_ in the XPS analysis. Figure 6c,d represent the total oxygen (O) concentration on the surface. Various components contribute to this concentration. Firstly, there is adsorbed contamination, which appears at a binding energy of 532.5 eV [57]. Secondly, aluminum in the hydroxide/oxyhydroxide state contributes to the O concentration, observed at a binding energy of 76.1 eV [58,59]. Thirdly, the aluminum oxide layer itself contributes to the O concentration, appearing at a binding energy of 531 eV [60]. Additionally, the presence of native aluminum oxides also contributes to the overall O concentration. 

These observations from the Al2p spectra and the total O concentration analysis provide further evidence of the presence and contribution of aluminum oxides, particularly Al_2_O_3_, on the corroded surface of the SLM AlSi10Mg parts, and no evidence of the presence of the Mg element was detected. The identification of different aluminum oxide species and their relative binding energies helps for understanding the composition and characteristics of the corrosion products on the surface.

### 3.2. Tensile Behavior of SLM Parts after Corrosion

**Figure 7:**
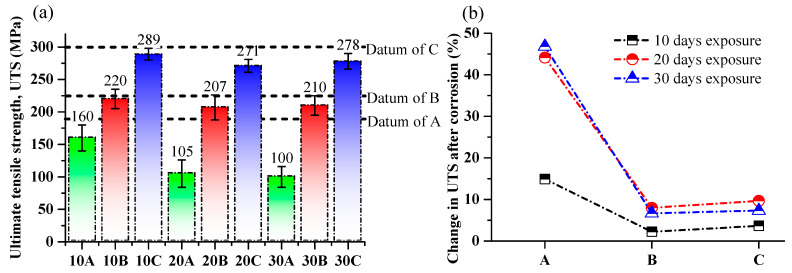
Ultimate tensile strength of as-corroded SLM parts in 3.5% NaCl solution with different exposure times: (**a**) UTS of as-corroded SLM parts and (**b**) change in UTS of as-corroded SLM parts.

The effect of corrosion on the tensile strength of SLM AlSi10Mg parts with different exposure times in a 3.5% NaCl solution was investigated (see Figure 7). All the samples were tested using a cross-head speed of 0.5 mm/min. The tensile strength values of the corroded samples were compared with the tensile strength of the as-built SLM parts mentioned as datum in Figure 6a (i.e., 188 MPa, 220 MPa, and 300 MPa for samples A, B, and C, respectively), while a percent change in tensile strength after corrosion with different exposure times is displayed in Figure 7b. The initial tensile strength measurements indicated that the 1 mm thick sample (A) had a tensile strength of 160 MPa after 10 days of exposure. Sample B, with a thickness of 2 mm, exhibited a higher tensile strength of 220 MPa, while sample C, with a thickness of 3 mm, had the highest tensile strength at 289 MPa. As the exposure time was increased to 20 days, a noticeable decrease in tensile strength was observed for all samples. Sample A experienced a significant reduction, with the tensile strength dropping to 105 MPa. Sample B also exhibited a decrease in tensile strength, but to a lesser extent, measuring 207 MPa. Sample C, despite the longer exposure time, maintained a relatively higher tensile strength of 271 MPa. Moreover, when the exposure time was further extended to 30 days, the tensile strength of the samples remained nearly unchanged, with only negligible differences observed. This can be attributed to the behavior of the corrosion layer. Initially, as the immersion time increased to 10 days, the corrosion layer experienced significant growth. However, as the immersion time extended to 20 days, the growth of the corrosion layer slowed down. This trend continued, and even after 30 days of immersion, the corrosion layer remained nearly unaffected. This observation is supported by both the mass loss measurements and XPS analysis, which confirmed the limited growth of the corrosion layer over time. The resistance to corrosion-induced deterioration in samples B and C can be attributed to the stabilization or deceleration of the corrosion process after an initial period of rapid growth.

The decrease in tensile strength of SLM parts after corrosion with different exposure times in a 3.5% NaCl solution can be attributed to several factors. Firstly, the loss of material due to dissolution and the formation of corrosion products can lead to a reduction in the overall mass and density of the parts also presented in Figure 3. Secondly, the breakdown of intermetallic phases [61] and the formation of pits and crevices [62] can create stress concentration points [63], which can further weaken the material (see Figure 2). Additionally, the presence of corrosion products can alter the microstructure of the alloy, affecting its mechanical properties [43,64,65]. The corrosion products may have different crystal structures and compositions compared to the original alloy, resulting in a less homogeneous and weaker material [43,66].

### 3.3. Variation in Hardness after Corrosion

**Figure 8:**
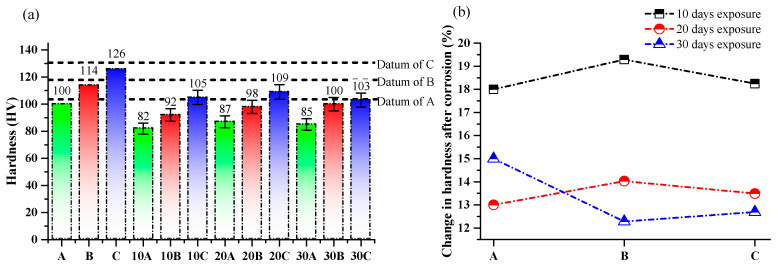
Hardness of as-corroded SLM parts in 3.5% NaCl solution: (**a**) hardness of as-corroded surface and (**b**) change in UTS of as-corroded SLM parts.

The hardness of the corroded SLM parts with different exposure times was measured using a 500 gm load and a 15 s dwell time on a LECO AMH 43 automatic hardness tester, following the guidelines of ASTM E-384. Figure 8 exhibits hardness values of the as-corroded SLM parts and is compared with the reference hardness values of the as-built SLM parts (i.e., 105 HV, 118 HV, and 131 HV for samples A, B, and C, respectively) mentioned as datum in Figure 8a. It can be seen that, after 10 days of exposure, sample A exhibits a hardness of 82 HV, sample B has a slightly higher hardness of 92 HV, and sample C has the greatest hardness at 109 HV. With an increase in exposure time to 20 days, the hardness of the corroded surfaces increased for sample A, sample B, and sample C. Sample A showed an increase to 87 HV, sample B increased to 98 HV, and sample C increased to 105 HV. When the exposure time was further extended to 30 days, the hardness values for all samples remained relatively similar. Sample A had a hardness of 85 HV, sample B measured 100 HV, and sample C attained 103 HV.

The data suggest that the hardness of the corroded surfaces does not show a significant overall trend of increasing or decreasing with increasing exposure time. However, it is worth noting that the hardness values for sample A and sample C exhibited a slight decrease at 30 days compared to their 20-day values. Figure 7b represents the percentage change in hardness values compared to the baseline values of as-built SLM parts (see Figure 8a). After 10 days of exposure, all the corroded samples exhibited a drop in hardness ranging from 18% to 19% compared to the as-built SLM parts. This significant decrease suggests that the corrosive environment had a notable impact on the hardness properties of the AlSi10Mg alloy. As the exposure time was extended to 20 days, the drop in hardness reduced slightly, ranging from 13% to 14%. Although the decrease was less pronounced compared to the 10-day exposure, it still indicated a significant reduction in hardness values. Interestingly, with a further increase in the exposure time to 30 days, the drop in hardness remained relatively similar, ranging from approximately 12% to 15% across the samples. This suggests that, beyond a certain exposure period, the corrosive environment may have reached a saturation point, resulting in a stabilization of the hardness drop.

It is worth noting that the decrease in hardness was more pronounced for the thinner sample (A) compared to the thicker samples (B and C). This can be attributed to the relatively higher surface-to-volume ratio of the thinner sample, making it more susceptible to the corrosive attack. The observed decrease in hardness can be attributed to the corrosive attack of the 3.5% NaCl solution on the AlSi10Mg alloy. Over time, the chloride ions from the solution penetrated the alloy surface, initiating corrosion and leading to the formation of corrosion products, which contributed to the reduction in hardness.

### 3.4. Fractured Surfaces of SLM Parts

**Figure 9:**
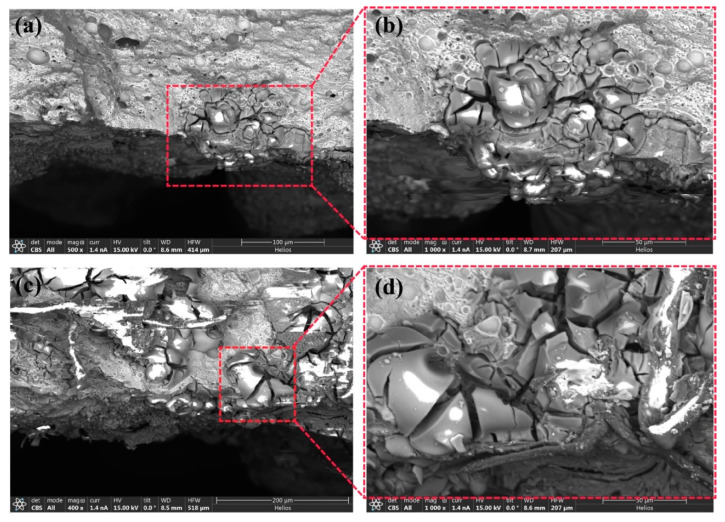
SEM images of fractured surface of as-corroded sample C: (**a**) 10-day exposure time, (**b**) magnified image of (**a**), (**c**) 30-day exposure time, and (**d**) magnified image of (**c**).

**Figure 10:**
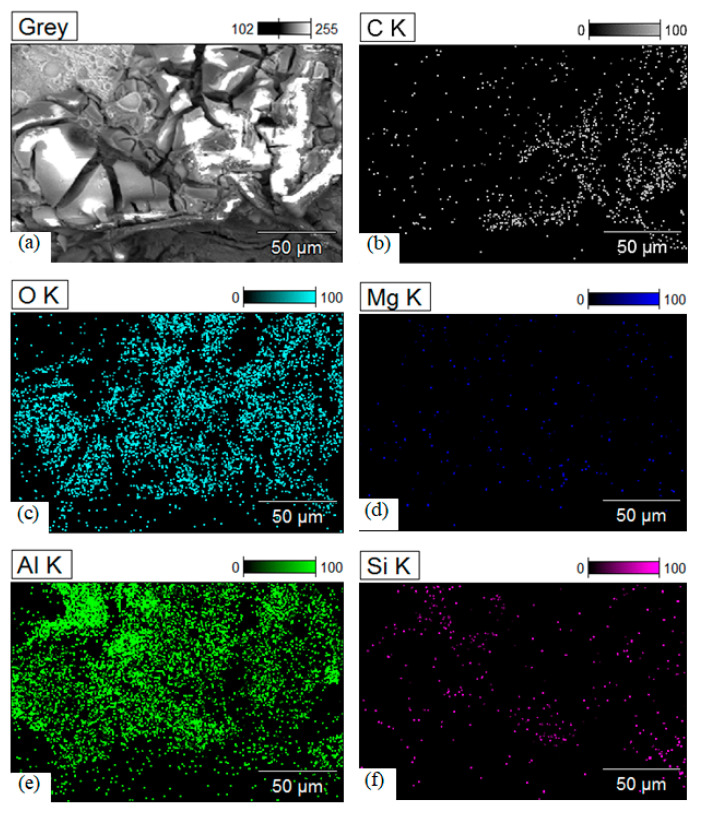
EDX maps of as-corroded SLM parts treated with 3.5% NaCl solution under 30-day exposure time. (**a**) Selected region for elemental mapping (**b**) Carbon (**c**) Oxygen (**d**) Magnesium (**e**) Aluminum, and (**f**) Siliconmaps.

The SEM scans of the as-corroded fractured SLM parts after tensile testing, treated with different exposure times in a 3.5% NaCl solution, provide valuable insights into the nature of the corrosion and the composition of the resulting surface. The formation of the corrosion product can be seen in Figure 9. A typical flowery pattern was observed in the corroded surface of sample C (Figure 9a) for a 10-day exposure time, which is magnified in Figure 9b for a clear visualization. It was also revealed from the SEM scans that the severity of corrosion compounds increased with the exposure time, which was ultimately responsible for the decrease in mechanical properties, as discussed previously. The SEM scans reveal that the fractured surface of the corroded parts contains corrosion products primarily composed of aluminum (Al) and oxygen (O). Additionally, the presence of silicon (Si), carbon (C), and magnesium (Mg) is also indicated in the EDX mappings of sample C (exposed for 30 days), as shown in Figure 10.

**Figure 11:**
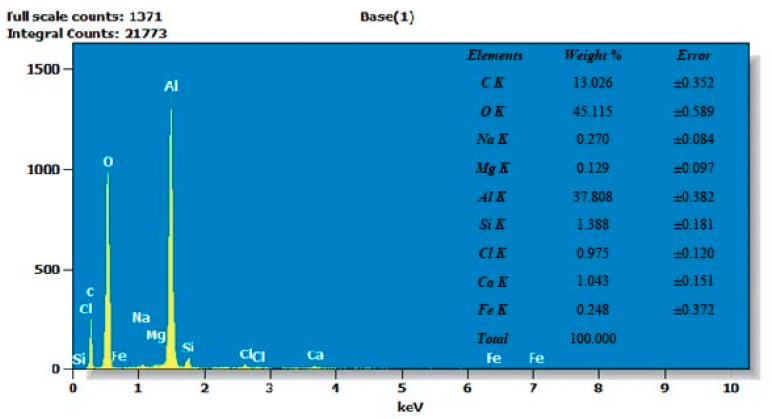
EDX peaks of as-corroded SLM parts.

The quantitative analysis extracted from the EDX analysis is shown in Figure 11. The corroded surface mainly contained 45 wt.% oxygen, 37.8 wt. % aluminum, 13 wt.% carbon, and other trace elements. The obtained results further support the observation that the corroded surface predominantly consists of aluminum oxide (Al_2_O_3_) compounds. The magnesium content was scarcely present as 0.129 wt.%, which indicated that magnesium was dissolved due to chloride action in the 3.5% NaCl solution [43]. This finding is also consistent with the XPS (X-ray photoelectron spectroscopy) results discussed previously, which provide evidence of the presence of Al_2_O_3_ on the corroded surface. The identification of Al_2_O_3_ as the main corrosion product aligns with the known behavior of aluminum alloys in corrosive environments. Aluminum readily forms a protective oxide layer, such as Al_2_O_3_, which acts as a barrier against further corrosion.

## 4. Conclusions

These findings demonstrate the importance of considering exposure time and sample thickness when evaluating the corrosion performance and mechanical properties of SLM AlSi10Mg parts in a 3.5% NaCl solution. The following points are the key issues highlighted in this research:

- In the corrosion test, increasing exposure time led to greater mass loss for all the samples. However, there was no apparent correlation between sample thickness and mass loss in the 3.5% NaCl solution.
- An illustration is provided that displayed porosities in all samples, with samples A and B showing a higher prevalence compared to sample C. Sample B exhibited smaller pore sizes, while sample C had minimal pore presence. The presence of more pores increased the surface area susceptible to corrosion, resulting in a more pronounced corrosion response.
- Surface examination revealed that the selective dissolution of Al and Mg was intensified with the increase in exposure time. The surface mainly contained Al_2_O_3_ oxide film, which became stable with the 30-day exposure time.
- The decrease in tensile strength of SLM parts after corrosion in a 3.5% NaCl solution can be attributed to material loss, and the formation of corrosion products. These factors contributed to weakened mechanical properties and reduced homogeneity in the material. A similar trend was also observed for the hardness of the as-corroded SLM parts.
- Significant changes in tensile strength were observed for samples A, B, and C with different exposure times. Sample A exhibited a drastic decrease in tensile strength, with reductions of 44.14% and 46.8% after 20 and 30 days of exposure, respectively, compared to the initial 10-day period (14.89%). Sample B showed a milder decline, with a 2.22% decrease after 10 days, escalating to 8% and 6.6% after 20 and 30 days, respectively. Sample C also experienced a decrease, with 3.66% at 10 days and 9.66% and 7.3% at 20 and 30 days, respectively. These findings indicate the susceptibility of the materials to corrosion and degradation, with sample A displaying the most severe changes, and samples B and C exhibiting lower severities but still significant reductions in tensile strength as the exposure time increased.
- Similarly, substantial reductions in hardness values were also observed with the increasing exposure time. Sample A displayed a drastic change, with hardness decreasing by 18% after 10 days, 13% after 20 days, and 15% after 30 days. Similarly, sample B exhibited a noticeable decline, with hardness decreasing by 19.29% after 10 days, 14.03% after 20 days, and 12.28% after 30 days. Sample C also recorded considerable reductions, with hardness decreasing by 18.25% after 10 days, 13.49% after 20 days, and 12.69% after 30 days of exposure time. These results highlight that prolonged exposure leads to significant decreases in hardness for all samples, indicating potential material degradation or alterations in the microstructure.

## Figures and Tables

**Table 1:** Operating parameters used for the preparation of SLM parts [39,40].

Technical/Operating Parameters	Values/Description
Laser power (kW)	0.32
Scan speed (m/s)	0.90
Hatch distance (mm)	0.08
Slice thickness (mm)	0.03
Beam focus diameter (mm)	0.08
Scanning strategy	67° with checkerboard
Building direction	Vertical
Building substrate plate	280.0 mm × 280.0 mm × 70.0 mm (L × W × H)

**Table 2:** Sample’s designation and its corresponding wall thickness used in the research.

S No.	Sample’s Designations	Wall Thickness (mm)
1	A	1
2	B	2
3	C	3

## Data Availability

Data are contained within the article.
